# The regulatory role of oxidative stress in the tooth movement process of rats with chronic fluorosis

**DOI:** 10.1186/s12903-025-06599-7

**Published:** 2025-08-20

**Authors:** Lingyan Lai, Wanxin Chen, Yue Hao, Bo Chen, Hua Yang

**Affiliations:** 1 Guizhou University of Traditional Chinese Medicine, Guiyang, 550000 China; 2 Department of Stomatology, Guizhou Provincial People’s Hospital, Guiyang, 550000 China; 3 Dalian University, Dalian, 116622 China; 4 Zunyi Medical University, Zunyi, 563000 China

**Keywords:** Orthodontic movement cycle, Fluorosis, Oxidative stress, Jawbone remodelling

## Abstract

**Background:**

Clinically, fluorosis patients exhibit delayed orthodontic tooth movement and compromised retention. Experimental studies in fluorosis-exposed rats demonstrate suppressed tooth movement, impaired periodontal angiogenesis, and downregulated VEGF/PI3K/AKT/eNOS signaling. Oxidative stress is critical in periodontal remodeling during orthodontic treatment, yet its role in fluorosis-related movement alterations remains unclear.

**Methods:**

Seventy 3-week-old Sprague-Dawley rats (body weight 60 ± 5 g) were randomly allocated into experimental groups. Ten rats served as a baseline group (0 day). The remaining 60 were randomized into three groups: control (C), orthodontic (O), and fluorosis orthodontic (FO), (*n* = 20 rats), subdivided into 3, 7, 14, and 21 days subgroups. C, O groups and blank baseline subgroup received purified water (fluoride < 0.08 mg/L, below the national standard of 1 mg/L), the FO group and fluorosis baseline subgroup drank 150 mg/L NaF water to establish a fluorosis model. After 3 months, orthodontic appliances were applied to O and FO groups. Fluoride accumulation (blood/urine), tooth movement rate and oxidative stress markers (SOD, CAT, MDA, and 8-OHdG) were analyzed.

**Results:**

1 FO group showed elevated blood/urine fluoride levels with C group (*P* < 0.05). Successful tooth movement was confirmed by interdental expansion.2 FO group exhibited slower tooth movement rate than O group (*P* < 0.05)0.3 Oxidative stress dynamics: Intergroup Difference: SOD and CAT levels were lowest in the FO group before 7 days, while MDA and 8-OHdG levels were highest in the FO group (*P* < 0.05), with differences narrowing in later stages.Intragroup Comparisons: SOD: C group: Levels initially increased, peaked at 14 days, and subsequently declined.O group: Levels consistently decreased over time.FO group: Levels exhibited a continuous upward trend.CAT: C group: Levels fluctuated in the early phase and sharply increased in the later phase.O group: Levels initially rose and then declined.FO group: Levels persistently increased throughout the study.MDA: C and FO groups: Levels continuously decreased.O group: Levels first increased, then decreased with fluctuations.8-OHdG: C group: Levels initially rose and later declined.O group: Levels fluctuated markedly.FO group: Levels first decreased and then slightly rebounded.

**Conclusion:**

Fluorosis inhibits early-stage tooth movement (3–7 days) through aggravated oxidative stress, with diminishing effects over time as compensatory antioxidant mechanisms emerge.

## Introduction

Under normal circumstances, fluorine is one of the essential trace elements for human life activities. It is beneficial to the conduction of the nervous system and the normal operation of the enzyme system. It can also promote the normal growth and development of teeth and bones, and prevent dental caries and osteoporosis [1]. However, chronic fluorosis can occur when the daily intake exceeds 1 mg.Chronic fluorosis is prevalent to varying degrees in more than 40 countries on five continents in the world, such as China, the United States, the United Kingdom, Australia, India and other countries, and it is one of the most widely distributed endemic diseases on the earth [1]. It is reported that more than 70 million people are affected by drinking water fluorosis in China, which is the main type of endemic fluorosis in China [2, 3]. In China, the prevalence of malocclusion reaches 72% (among both children and adults), theoretically indicating a potential demand population exceeding 1 billion. Approximately 20–40% of patients with mild to moderate dental fluorosis require orthodontic treatment. Therefore, it is imperative to investigate and differentiate the patterns of tooth movement and tissue remodeling in orthodontic patients with dental fluorosis, which will provide experimental evidence and research foundation for developing specific clinical strategies.

Periodontal tissues, serving as a crucial interface connecting systemic and local bodily environments, perform essential functions including mechanical support, barrier protection, and immune defense. These tissues maintain dynamic interactions with systemic metabolism, immune responses, and inflammatory reactions, constituting a pivotal nexus for systemic-local biological interplay. Studies have demonstrated that patients with systemic diseases such as diabetes mellitus, rheumatoid arthritis, and osteoporosis exhibit increased susceptibility to periodontitis. Epidemiological investigations reveal compromised periodontal health status in populations residing in fluorosis-endemic areas, where individuals with chronic fluorosis manifest higher prevalence and severity of gingivitis, periodontitis, and periodontal bone loss.

Fluoride is primarily absorbed through the digestive tract in the form of soluble fluoride compounds (e.g., NaF, CaF₂), transported via systemic circulation, and preferentially deposited in mineralized tissues. Approximately 50-60% of absorbed fluoride accumulates in bones and teeth, with eventual renal excretion. These metabolic processes induce systemic damages of varying severity, among which skeletal fluorosis manifests as the most immediate and severe manifestation of chronic fluorosis. The biological basis of orthodontic treatment lies in periodontal tissue remodeling. Bone remodeling equilibrium serves as both a necessary and sufficient condition for achieving orthodontic tooth movement and stable reconstruction.From a broad perspective, all endogenous and exogenous factors influencing bone metabolic equilibrium may potentially modulate orthodontic tooth movement progression. Chronic fluorosis—a lifelong systemic toxicosis predominantly manifesting as skeletal fluorosis—therefore establishes pathophysiological pathways affecting periodontal tissue remodeling. Our preliminary investigations reveal that fluorotic rats exhibit microarchitectural transition toward lamellar bone patterns in alveolar structures. Characteristic periodontal osteogenic-osteoclastic imbalance manifests as temporally distinct upregulation of Runx2, BMP-2, and ALP during osteogenic differentiation and maturation phases, concomitant with altered tooth displacement biomechanical signatures.

The pathogenesis of fluorosis involves multifaceted mechanisms including protein alteration hypothesis, nucleic acid damage theory, oxidative stress paradigm, signal transduction pathway dysregulation, and lipid metabolic disorder hypothesis. Among these, the oxidative stress hypothesis has garnered substantial scientific consensus and research focus.Extensive studies demonstrate elevated total oxidative status (TOS) and diminished total antioxidant capacity (TAC) in sera of chronic fluorosis patients [4]. The core mechanism resides in fluoride ions (F⁻) disrupting mitochondrial electron transport chain (ETC) functions, resulting in excessive reactive oxygen species (ROS) generation while simultaneously suppressing endogenous antioxidant defense systems. This redox imbalance activates inflammatory cascades, triggering lipid peroxidation, protein carbonylation, and DNA oxidative damage, ultimately culminating in apoptotic cascades and histopathological alterations [5–11]. Fluorosis (dental fluorosis and skeletal fluorosis) represents not only the earliest manifestations but also the most characteristic clinical features of chronic fluoride poisoning.Clinical observations indicate that the orthodontic treatment duration for patients with dental fluorosis is longer than that for non-fluorosis patients, and achieving tooth movement is also more difficult [12–14]. Research shows that moderate oxidative stress may be involved in the biological regulation of orthodontic tooth movement. Possible factors include dental materials, hypoxia in the microenvironment of periodontal tissues caused by tooth movement, and enhanced inflammatory responses [15–22]. Current research has documented limited exploration into how oxidative stress levels in fluoride-intoxicated organisms may influence the biomechanical characteristics of orthodontic tooth movement and periodontal tissue remodeling, particularly regarding their phenotypic manifestations and underlying mechanisms.

Based on these findings, our study hypothesizes that oxidative stress modulation under chronic fluorosis conditions may exert regulatory involvement in physiological orthodontic tooth movement. This hypothesis will be systematically validated through the following preclinical investigations employing established animal models.

## Methods

**Fig. 1:**
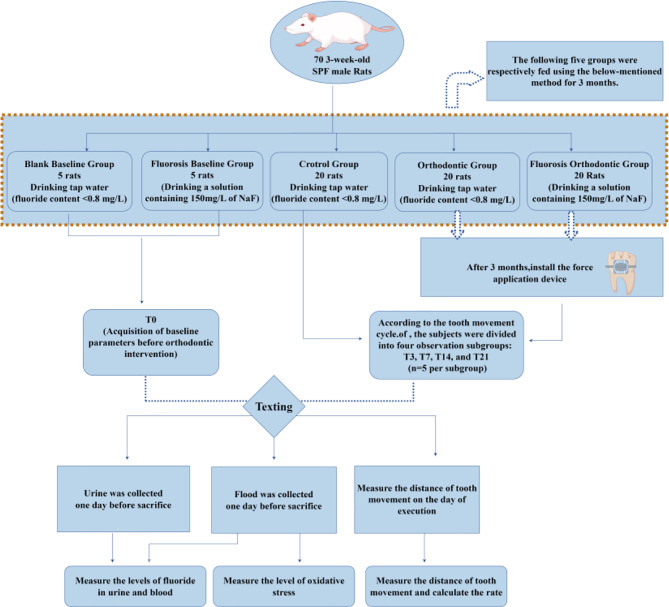
Experiment flow chart

A schematic representation of the experimental workflow is provided in Fig. 1.

### Experimental subjects and grouping

A total of 70 3-week-old SPF male Sprague-Dawley (SD) rats with body weight 55–65 g(provided by Experimental Animal Center of Guizhou Medical University), certificate number SCXK (Xiang) 2019-0014, were fed in the Experimental Animal Center of Guizhou Medical University [SYXK (Guizhou) 2018-0001], humidity 55-60%, temperature 22–24 ℃, alternate lighting environment for 12 h.University of Science and Technology Experimental Animal Ethics Committee examined and approved (IACUC number: NO.2000890), according to the principle of 3R used by experimental animals to provide humanitarian care.After acclimating the laboratory rats for one week, 60 rats were randomly divided into three groups using a random number table method: blank control group (C Group), orthodontic control group (O Group), and fluorosis orthodontic group (FO Group), with 20 rats in each group. Based on the human orthodontic tooth movement cycle, four observation time points were set: 3 days (T3), 7 days (T7), 14 days (T14), and 21 days (T21). Each time point corresponds to a subgroup (5 rats per subgroup). The remaining 10 rats were set as the 0-day (T0) baseline group (including blank baseline subgroup and fluorosis baseline subgroup, with 5 rats per subgroup) Table 1.

**Table 1:** The experimental group of rats(unit: number)

Time Group	0 day	3 days	7 days	14 days	21 days
Control Group (C Group)	5	5	5	5	5
Orthodontic Group(O Group)		5	5	5	5
Fluorosis Orthodontic Group (FO Group)	5	5	5	5	5

### Main experimental reagents

Thunder magnetic composite fluorine electrode (CSB-F-2 type, China Changsha Yitian Experimental Instrument Co., LTD. Spring Flower Production Center), sodium fluoride (Sigma Aldrich), Total superoxide dismutase (SOD) kit (Nanjing Jiancheng Bioengineering Institute), malondialdehyde (MDA) kit (Nanjing Jiancheng Bioengineering Institute), catalase (CAT) kit (Nanjing Jiancheng Bioengineering Institute), 8-hydroxy-deoxyguanosine(8-OHdG) ELISA kit (Jiangsu Jingmei Biotechnology Co., LTD.).

### Modeling

The fluorosis model was established by drinking a sodium fluoride-containing aqueous solution. C group O group and blank baseline subgroup regularly consumed tap water (fluoride content < 0.8 mg/L, lower than the national standard drinking water fluoride content of 1 mg/L). The concentration used to establish the fluorosis model was 150 mg/L, and this concentration and water consumption were set based on previous studies [23, 24]. The fluoride content was quantified at < 0.6 mg/kg in controls, whereas the fluorosis model group demonstrated a calculated daily fluoride intake of 45.03 µg/g body weight (equivalent to 45.03 mg/kg BW/day) through precise metabolic cage monitoring The success of the model was judged by the presence of dental fluorosis and the continuous increase of blood fluoride and urine fluoride.

After feeding fluoride for 3 months, orthodontic group and fluorine-dyed orthodontic group began to build orthodontic tooth moving model according to the method of reinforcement in previous repeated experiments.Rats were anesthetized by injecting with 2% sodium pentobarbital (3 mL/kg) at the midline of the abdomen and then fixed in the supine position.The labial side of the anterior teeth is bonded and polished in advance to form brackets of appropriate size.Insert a 0.25 mm ligature wire between the distal of the maxillary first molar and the mesial of the second molar, making the ligature wire closely adhere to the cervical part of the teeth, and then insert the nickel-titanium pull spring into the ligature wire.Use a dynamometer to set the force application unit of the pull spring to 70 g. Tie the bilateral pull springs to the anterior tooth brackets with ligature wires, and fix them with green glue light irradiation to establish a rat orthodontic tooth movement model.

### Tissue harvesting and preservation

The baseline group was euthanized via intraperitoneal injection of 2% pentobarbital sodium (3 mL/kg body weight) at 0 days post-force application for tissue harvesting. Subsequent groups were processed using the same protocol at 3, 7, 14, and 21 days following force application.

Urine sample collection: Rats underwent a 24-hour fasting period (ad libitum water withdrawal) prior to euthanasia and were individually housed in metabolic cages. Urine samples were collected over a 24-hour period and immediately stored at -80 °C pending biochemical analysis.

Blood sample collection: Following intraperitoneal anesthesia, cardiac puncture was performed for terminal blood collection. Whole blood was allowed to clot at room temperature (23 ± 1 °C) for 2–3 h prior to centrifugation at 503 × g (equivalent to 3000 rpm with 5 cm rotor radius) for 10 min. Serum aliquots were cryopreserved at − 80 °C pending biochemical analysis.

### Determination of fluoride in urine and blood

Urinary fluoride: According to WS/T 89-2015,Lanthanum fluoride single crystals are selective for fluoride ions, and there is a potential difference (membrane potential) between different concentrations of fluoride solutions on either side of the lanthanum fluoride single crystal membrane of the fluoride electrode. The size of the membrane potential is related to the activity of fluoride ions in the solution. Within a certain activity range, the electric potential of the electrochemical cell composed of the fluoride electrode and the calomel electrode is linear with the logarithm of the fluoride ion activity, so that the concentration of fluoride ions in urine can be determined. And the detection limit of this method was 0.1 mg/L, and the recovery rate was 98.9-100.5%.

Blood fluoride: According to WS/T 212–2001 “Ion selective electrode method for determination of fluoride in serum”,determination of inorganic fluoride concentration in serum using fluoride ion-selective electrode method similar to urinary fluoride determination.And the detection limit of the method was 0.012 µg /mL, and the recovery rate was 96.7% ~ 101.2%.

### Detection of oxidative stress level

After anesthesia, blood was collected via cardiac puncture, and rat blood samples were obtained for oxidative stress analysis.

The activity of superoxide dismutase (SOD) was measured using the WST-1 assay, a water-soluble thiazole salt that reacts with the superoxide anion (O₂-) produced by Xanthine Oxidase (XO) to form a water-soluble yellow formazan dye.SOD catalyses the disproportionation of superoxide anion to inhibit the formation of formazan. SOD can catalyse the disproportionation of superoxide anion and thus inhibit the generation of filth. Therefore, SOD activity is negatively correlated with the amount of filth production, which can be calculated by colourimetric analysis (450 nm).

The viability of catalase (CAT) was measured using the ammonium molybdate method.CAT catalyses the decomposition of hydrogen peroxide (H₂O₂) into water and oxygen. In the presence of ammonium molybdate, H₂O₂, which is not decomposed by CAT, reacts with ammonium molybdate to form a yellow complex with an intense absorption peak at 405 nm, and its absorbance value is proportional to the concentration of H₂O₂. The amount of H₂O₂ catalytically decomposed by CAT can be calculated by measuring the amount of H₂O₂ remaining in the reaction system, thus reflecting the CAT activity.

The malondialdehyde (MDA) content using thiobarbituric acid (TBA).MDA is one of the products of lipid peroxidation, which can react with TBA under acidic conditions to form a red MDA-TBA adduct, which has a maximum absorption peak at 532 nm. The absorbance can be determined by colourimetric method to quantitatively analyse the MDA content.

Serum 8-hydroxy-2’-deoxyguanosine (8-OHdG) content was determined by enzyme-linked immunosorbent assay to detect oxidative stress in rats.

### Distance and speed of tooth movement

Following orthodontic force application, mesial movement distances of the first molars were quantitatively assessed at designated observation time points (T0, T3, T7, T14, T21) using digital model superimposition.Place the measuring end plane of the positioning caliper close to the midpoint of the mesial gingival margin of the maxillary first molar, and the movable end of the positioning caliper close to the distal - lingual - gingival angle of the ipsilateral maxillary central incisor. Read the value using a vernier caliper. (It can be accurate to 0.01 mm.) The two measurements were carried out by the same person, and the measurement was repeated for three times for each specimen to calculate the average value. The difference between the two means was the distance M (mm) of the tooth movement of the rat after stress.

The segmental displacement rate of tooth movement was divided into four periods: T_0-3 d_, T_3-7 d_, T_7-14 d_ and T_14-21 d_. The corresponding segmental displacement was M_0-3 d_, M_3-7 d_, M_7-14 d_ and M_14-21 d_, respectively, in mm, and the corresponding rates were V_0-3 d_, V_3-7 d_, V_7-14 d_ and V_14-21 d_, respectively, in mm/d.

### Statistical analysis

SPSS 26.0 statistical software was used for statistical analysis of the data. If the measurement data conform to normal distribution, it is expressed by mean ± standard deviation; if it does not conform to normal distribution, it is expressed by median (interquartile). One-way analysis of variance (ANOVA) was used to compare the differences between groups with normal distribution and homogeneity of variance. LSD test was used for homogeneity of variance and Tamhane T2 test was used for heterogeneity. Nonparametric test is used for indexes that do not conform to normal distribution. The test level was α = 0.05, and < 0.05 was considered statistically significant.

## Results

### Model identification

#### Fluorosis model identification

**Fig. 2:**
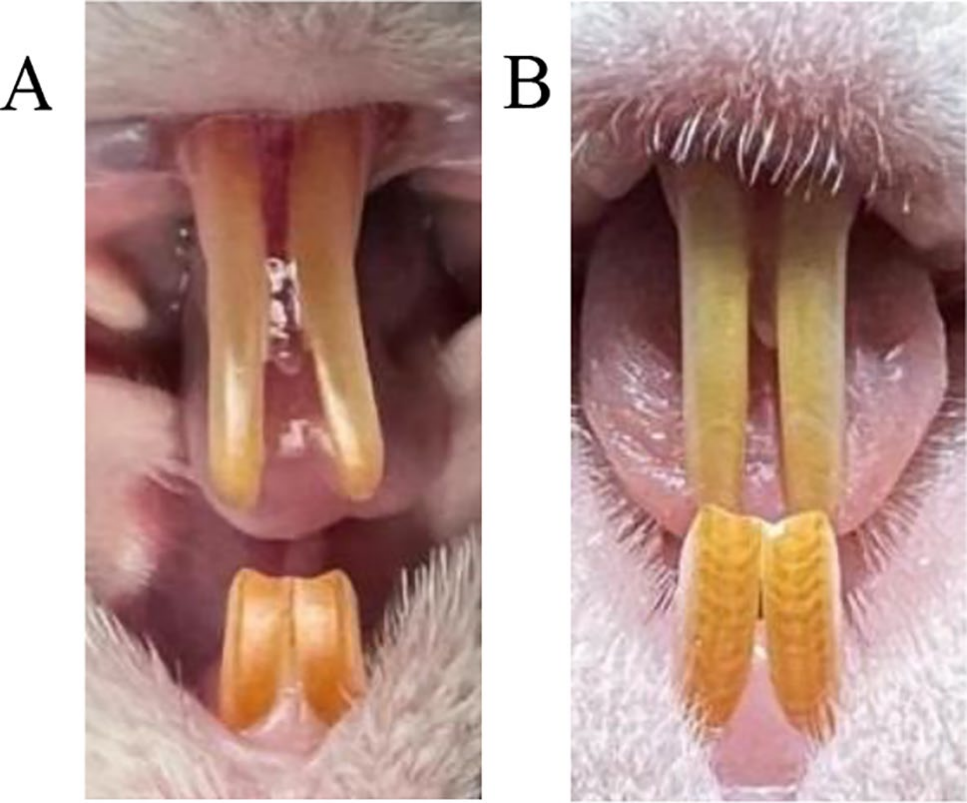
The appearance of dental fluorosis in rats. Note: **A** represents the rat in the **C** group and the O group. The teeth of the rats are all in a physiological state with smooth, shiny surface and translucent shape. **B** represents the rat after 3 months of fluoride exposure. The teeth show tarnish, brown and white stripes, with chalky stripes

After 3 months of fluoride exposure, rats exhibited symptoms of dental fluorosis. The urine fluoride content in the FO group (4.919 ± 0.593) was higher than that in the other groups (1.712 ± 0.467), and the blood fluoride content in the FO group (0.097 ± 0.017) was higher than that in the other groups (0.042 ± 0.004) (both *P* < 0.05), indicating that the chronic fluorosis animal model was successfully replicated Fig. 2.

#### Identification of normal tooth movement model

**Fig. 3:**
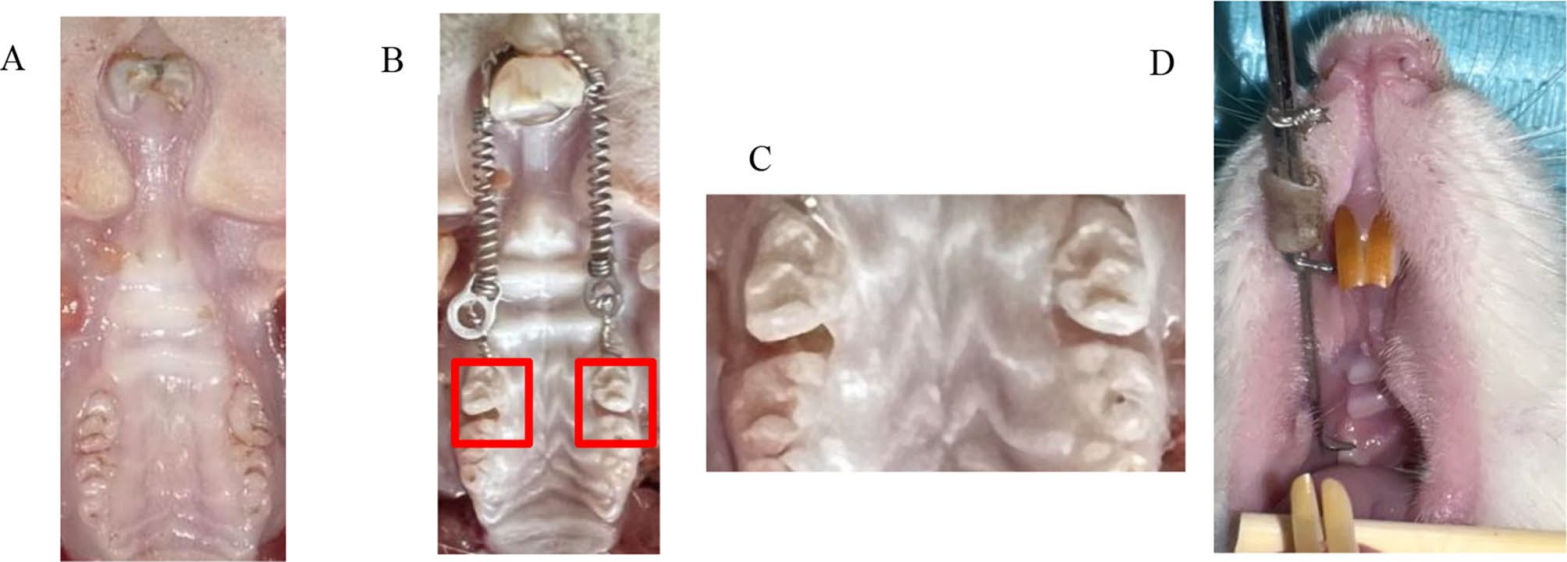
Changes in rat orthodontic tooth moving adjacent molar spaces. Note: **A** represents the rats in the blank group, **B** represents the rats with orthodontic force applied, showing gaps between the molars, **C** is a local magnification of the gaps between the molars, and **D** is using positioning caliper to neasure

After the orthodontic reinforcement device was placed in O group and FO Group, the progressive movement gap between the first and second molar teeth of the bilateral upper dentition was observed, indicating that the orthodontic tooth movement model was successfully replicated Fig. 3.

### Effects of fluoride exposure on tooth movement cycle of rats

**Fig. 4:**
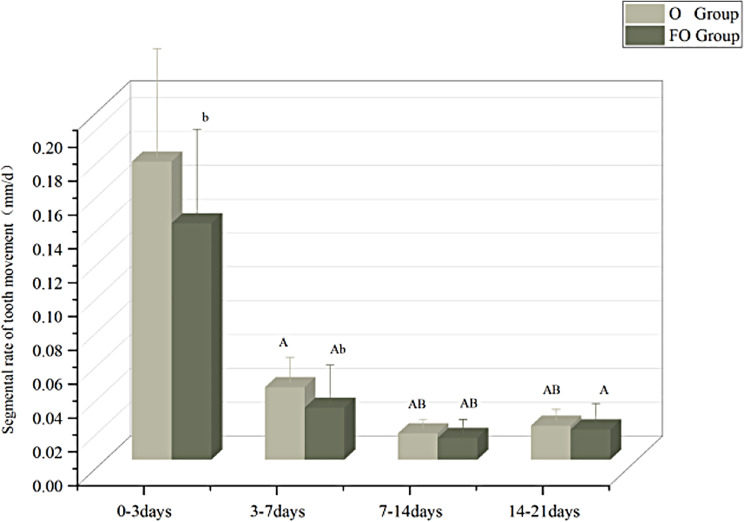
The segmental rate of tooth movement. Note: b: compared with the O group, *P* < 0.05; **A**: compared with 0 day, *P* < 0.05; **B**: compared with 3 days, *P* < 0.05

FO Group and O group rats exhibit a similar segmented rate of tooth movement, which shows a high degree of similarity to the biological cycle of human tooth movement: “initial phase - lag phase - linear movement phase.“That is, days 0–3 represent the initial phase of tooth movement in experimental rats, days 3–14 represent the lag phase, and days 14–21 represent the linear movement phase. When comparing the F and O groups, the tooth movement rate slows down in the FO Group Fig. 4.

### Combined effect of excessive fluoride and orthodontic force on oxidative stress in rats

#### SOD

Compared with C Group, the SOD activity in O Group and FO Group was decreased, and the differences were statistically significant in O Group at T (14, 21) and in FO Group at all time points (except T7) (*P* < 0.05).

**Fig. 5:**
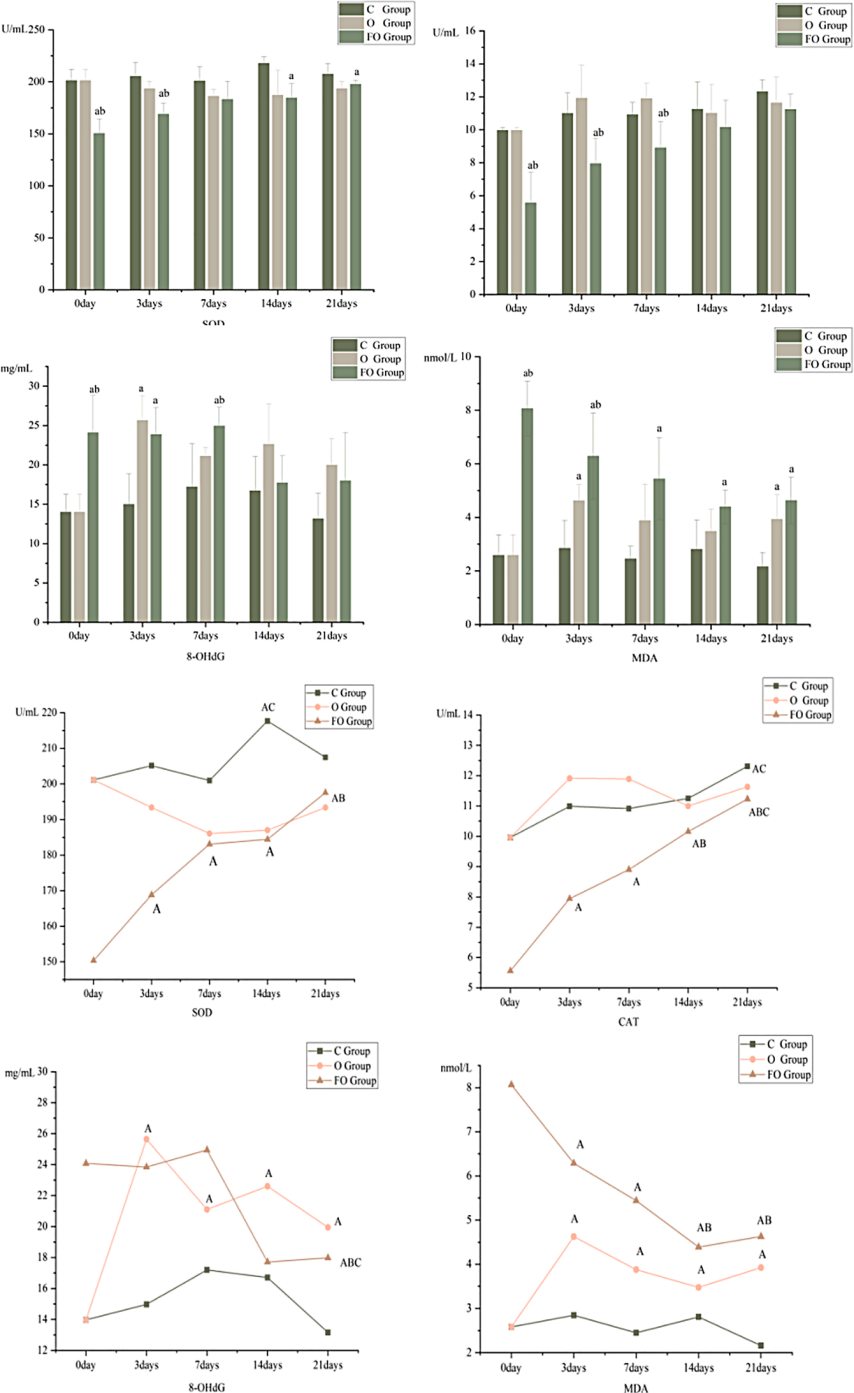
Changes in oxidative stress levels at different time points in each O Groupf rats. Note: **a**: compared with the C group, *P* < 0.05; **b**: compared with the O group, *P* < 0.05; **A**: compared with 0 day, *P* < 0.05; **B**: compared with 3 days, *P* < 0.05; **C**: compared with 7 days, *P* < 0.05; **D**: compared with 14 days, *P* < 0.05

Compared with O Group, in the early stage of tooth movement T (0, 3), the SOD activity in FO Group decreased, and the difference was statistically significant (*P* < 0.05) Fig. 5.

#### CAT

Compared with C Group, there was no statistically significant change in CAT activity in O Group. Compared with C Group and O Group, the CAT activity in FO Group decreased at T (0, 3, 7), and the differences were statistically significant (*P* < 0.05) Fig. 5.

#### MDA

Compared with C Group, the MDA content in O Group and FO Group increased. Among them, in O Group at T (3, 21), and in FO Group at all time points (T0–21), the differences were statistically significant (*P* < 0.05).

Compared with O Group, the MDA content in FO Group increased at T(0, 3), and the difference was statistically significant (*P* < 0.05) Fig. 5.

#### 8-OHdG

Compared with C Group, the 8-OHdG content in O Group and FO Group increased at T3 and T(0, 3, 7), respectively, and the differences were statistically significant (*P* < 0.05).

Compared with O Group, the 8-OHdG content in FO Group increased at T(0, 7), and the difference was statistically significant (*P* < 0.05) Fig. 5.

### The Temporal change effect of oxidative stress level in fluoride poisoning rats

#### SOD

In C Group, the expression of SOD increased over time, reaching a peak at T14, which was higher than the levels at previous time points (*P* < 0.05), and then gradually decreased. However, within the observation period, a statistically significant trough value has not yet appeared.

In O Group, the SOD level decreased and then increased gradually over time. There was no statistically significant difference when comparing different time points. Compared with C Group, the time-activity effect curve in the orthodontic group was more gentle.

In FO Group, the expression of SOD gradually increased over time, reaching a peak at T21, which was higher than that at T(0, 3), and the difference was statistically significant. Compared with O Group, the time-activity effect curve in FO Group was more significant, and the change trend was similar starting from the 7 days Fig. 5.

#### CAT

In C Group, the CAT activity increases slowly over time, reaching a peak at T21, which is higher than that at T(0, 7), and the difference was statistically significant (*P* < 0.05).

In O Group, the CAT activity curve fluctuates slightly, but there is no statistically significant difference when comparing each time point pairwise. Compared with C Group, the time-activity effect curve of O Group is similar to that of C Group.

In FO Group, the expression of CAT rapidly increases over time. The CAT content at T21 is higher than that at T(0, 3, 7), T14 is greater than T(0, 3, 7), and T3 is higher than T0 (*P* < 0.05). Compared with the O group, the time-activity effect curve of CAT in the FO Group is steeper Fig. 5.

#### MDA

In C Group, the expression of MDA is stable, and there is no statistically significant difference between any two time points.

In O Group, MDA increases early and reaches its peak at T3, and then decreases slowly. However, there is no statistically significant difference compared with T3. Moreover, MDA at all time points is higher than that at T0, and the difference is statistically significant (*P* < 0.05). Compared with C Group, the time-concentration effect curve of MDA in O Group is similar to that in C Group.

In FO Group, the MDA content decreases over time. The MDA content at T21 is less than that at T(0, 3), the MDA content at T14 is less than that at T(0, 3), and the MDA content at T(3, 7) is less than that at T0. The differences are statistically significant (*P* < 0.05). Compared with the O group, the changing trend of the FO Group is similar after 3 days Fig. 5.

#### 8-OHdG

In C Group, the expression of 8-OHdG is stable, and there is no statistically significant difference between each time point when compared pairwise.

In O Group, the expression curve of 8-OHdG showed a bimodal change, reaching a peak at 3 days, and the peak was higher than that of T0. The difference was statistically significant (*P* < 0.05), after which the curve fluctuated, but there was no statistically significant difference between the pre-and post-time points, only above T0 level (*P* < 0.05).

In FO Group, the expression of 8-OHdG fluctuates within a small range. There is a statistically significant difference that T21 is less than T(0, 3, 7) (*P* < 0.05). Compared with the time-content effect curve of O Group, the change trend is completely opposite Fig. 5.

## Discussion

Orthodontic treatment relies on balanced remodeling of periodontal bone tissue [25]. Systemic diseases, nutritional status, and other factors may adversely interfere with periodontal bone remodeling during orthodontic tooth movement, thereby affecting the tooth movement response. To date, no studies have reported on the relationship between oxidative stress levels in chronic fluorosis rats and the rate/duration of orthodontic tooth movement. This study aims to investigate the association between oxidative stress levels and the rate/periodic changes of orthodontic tooth movement under chronic fluorosis conditions.

The fundamental biological research in orthodontic treatment aims to elucidate the mechanisms by which mechanical signals generated by orthodontic forces are translated into biological responses in teeth and their supporting tissues [26–27]. Orthodontic forces are recognized as a form of physical stress applied to the periodontal ligament [28]. Under mechanical loading, various inflammatory mediators (i.e., cytokines) associated with aseptic inflammation are released within the periodontal ligament following the application of orthodontic force. These mediators initiate a cascade reaction in periodontal tissues, leading to systemic alterations in oxidative stress levels, which subsequently drive tissue remodeling and tooth movement [17–18]. The findings of this study align with previous research conclusions, while further demonstrating that oxidative stress levels within the body actively participate in the cyclical variations of tooth movement during orthodontic procedures. In this study, tooth movement in rats generally adhered to a classical triphasic pattern characterized by “rapid-slow-rising” stages. Notably, the differential rates of tooth movement across these three phases exhibited corresponding variations in oxidative stress levels.During the rapid tooth movement phase, lipids and DNA within the biofilm are disrupted, indicating that free radicals are introduced into the body under mechanical stimulation. The organism converts mechanical signals into biological signals, activating the oxidative stress response to promote bone remodeling and accelerate tooth movement. Subsequently, the antioxidant enzyme SOD begins scavenging excess free radicals, preventing oxidative damage caused by their accumulation. By 21 days, lipid peroxidation recurs in the biofilm, reactivating oxidative stress and initiating a “second rapid movement phase.” The temporal expression patterns of MDA and 8-OHdG in the orthodontic group paralleled those in the control group, suggesting that oxidative stress activation during tooth movement is not indicative of oxidative damage but rather represents a beneficial oxidative stress response. This phenomenon may arise from antioxidants triggering endogenous adaptive stress responses and inducing intrinsic defense mechanisms as potential initiators of tooth movement. In the future, integrating multi-omics technologies with intelligent diagnostic and treatment systems could enable precise redox regulation in orthodontic therapy, optimizing treatment outcomes through targeted oxidative stress modulation. Chronic fluorosis is a systemic disease characterized primarily by skeletal-phase damage as its pathological hallmark. Previous investigations from our research team revealed upregulated expression of osteogenic and osteoclastic biomarkers, including Runx2, BMP-2, Rankl, and OPG, accompanied by delayed orthodontic tooth movement velocity in fluorosis-exposed rats. Oxidative stress not only constitutes a crucial mechanism underlying fluorotoxicity but also functions as an initiator of orthodontic tooth movement. Our preliminary experiments have demonstrated elevated expression of the PI3K/Akt pathway, a downstream effector of oxidative stress responses [29, 30]. Therefore, we hypothesize that chronic fluorosis may impair orthodontic tooth movement through oxidative-antioxidant imbalance caused by altered systemic oxidative stress levels, resulting in oxidative damage that interferes with normal orthodontic processes.Chronic fluorosis primarily affects the temporal phase distribution (rate allocation) within the orthodontic tooth movement cycle [29]. In this study, we observed that fluoride exposure induces higher oxidative stress levels compared to physiological orthodontic conditions. Fluorosis generally reduces antioxidant enzyme content, enhances peroxidation product expression, activates oxidative stress, and inhibits orthodontic tooth movement. On 3 days of the rapid tooth movement phase, fluoride supplementation caused significant reductions in the activities of antioxidant enzymes SOD and CAT. As the primary defense of the biological antioxidant system, SOD catalyzes the decomposition of exogenously introduced superoxide radicals into oxygen and hydrogen peroxide. CAT, functioning as a terminal oxidase, subsequently breaks down hydrogen peroxide in vivo. When free radical production exceeds antioxidant capacity or when the antioxidant system becomes compromised, the oxidative-antioxidative equilibrium is disrupted, leading to oxidative stress-induced tissue damage [31, 32]. The persistent low expression of CAT from days 3 to 7 may stem from differential sensitivity of antioxidants to fluoride exposure. Oxidative agents are recognized as critical biological signals for environmental perception, operating through mechanical cellular stress and hemodynamic alterations generated under various physiological and pathological conditions [33, 34]. Fluoride, a potent oxidant [35], not only facilitates increased reactive free radical attacks on biological systems—inducing oxidative damage and activating oxidative stress—but also directly suppresses antioxidant enzyme activity while prematurely triggering endogenous defense mechanisms. 8-OHdG a biomarker strongly associated with oxidative damage in chronic diseases, has been validated for assessing oxidative injury in periodontal inflammation [36–38]. In this study, fluoride exposure induced abnormally elevated 8-OHdG levels, suggesting potential periodontitis in fluorotic rats, a finding consistent with epidemiological surveys [39]. Compared to the 3 days surge in malondialdehyde (MDA), the delayed 8-OHdG elevation at 7 days may stem from fluoride’s preferential impact on biofilm lipids, leading to MDA hyperaccumulation and subsequent DNA damage. As the repository of genetic information, DNA integrity governs disease pathogenesis. The time-dependent 8-OHdG fluctuations induced by excessive fluoride intake inversely correlate with orthodontic responses, likely attributable to fluoride’s cumulative deposition-release dynamics and compensatory physiological regulatory mechanisms.

Our preliminary experimental data demonstrated suppressed expression of osteogenic markers (Runx2, BMP-2) and angiogenic regulators (VEGF, eNOS), concomitant with elevated Rankl levels during the acceleration (3 days) and deceleration phases (7 days) of orthodontic tooth movement, confirming fluoride-induced non-physiological bone remodeling. Integrated with current findings, we postulate that oxidative stress exerts inhibitory effects on early-stage tooth movement in fluoride-rich environments, potentially through direct or indirect modulation of maxillofacial bone metabolic dysregulation. Nevertheless, the precise regulatory axis requires rigorous elucidation. Chronologically, the toxicological impact of fluoride progressively attenuates, with compensatory negative feedback mechanisms ultimately restoring systemic homeostasis.”

## Conclusion

Our mechanistic study demonstrates that chronic fluorosis-induced elevation of systemic oxidative stress dynamically regulates orthodontic tooth movement, primarily through redox-mediated inhibition of osteoclastic bone resorption, with significant suppressive effects observed specifically during the early remodeling phase (3–7 days).

### Study limitations

This study has certain limitations. While we focused on systemic oxidative stress levels, the alterations in local oxidative stress within the mandibular tissues require further investigation. Currently, there is almost no research on oxidative stress responses in rat mandibular tissues. This gap may arise because the rat mandible, as a typical lamellar bone structure, exacerbates the mineralization barrier effect, hindering the release or detection of biomarkers. Additionally, the lower metabolic activity of lamellar bone may lead to differential rates of oxidative stress production and clearance, resulting in shorter biomarker half-lives or reduced concentrations, thereby increasing detection challenges for conventional methods. Future studies could employ advanced techniques such as spatial metabolomics and nanoprobe-based detection to address these limitations.

## Data Availability

Data is provided within the manuscript or supplementary information files.

## Abbreviations

NaF: Sodium Fluoride
C group: Control Group
O group: Orthodontic Group
FO Group: Fluoride-Stained Orthodontic Group
SOD: Superoxide Dismutase
MDA: Malondialdehyde
CAT: Catalase
SD: Sprague-Dawley
VEGF: Vascular Endothelial Growth Factor
eNOS: Endothelial Nitric Oxide Synthases
